# Structural and functional characterization of a multi-domain GH92 α-1,2-mannosidase from *Neobacillus novalis*

**DOI:** 10.1107/S2059798323001663

**Published:** 2023-04-18

**Authors:** Bartłomiej M. Kołaczkowski, Olga V. Moroz, Elena Blagova, Gideon J. Davies, Marie Sofie Møller, Anne S. Meyer, Peter Westh, Kenneth Jensen, Keith S. Wilson, Kristian B. R. M. Krogh

**Affiliations:** a Department of Science and Environment, Roskilde University, Universitetsvej 1, Building 28, 4000 Roskilde, Denmark; b Novozymes A/S, Biologiens Vej 2, 2800 Kongens Lyngby, Denmark; c York Structural Biology Laboratory, Department of Chemistry, University of York, York YO10 5DD, United Kingdom; d Department of Biotechnology and Biomedicine, Technical University of Denmark, Building 224, 2800 Kongens Lyngby, Denmark; Station Biologique de Roscoff, France

**Keywords:** glycans, α-mannosidases, *Neobacillus novalis*, glycoside hydrolase family 92, carbohydrate-binding module family 32

## Abstract

Many secreted eukaryotic proteins are N-glycosylated with oligosaccharides composed of a high-mannose N-glycan core and, in the specific case of yeast cell-wall proteins, an extended α-1,6-mannan backbone carrying a number of α-1,2- and α-1,3-mannose substituents of varying lengths. α-Mannosidases from CAZy family GH92 release terminal mannose residues from these N-glycans, providing access for the α-endomannanases, which then degrade the α-mannan backbone. Most characterized GH92 α-mannosidases consist of a single catalytic domain, while a few have extra domains including putative carbohydrate-binding modules (CBMs). To date, neither the function nor the structure of a multi-domain GH92 α-mannosidase CBM has been characterized. Here, the biochemical investigation and crystal structure of the full-length five-domain GH92 α-1,2-mannosidase from *Neobacillus novalis* (*Nn*GH92) with manno­imidazole bound in the active site and an additional mannoimidazole bound to the N-terminal CBM32 are reported. The structure of the catalytic domain is very similar to that reported for the GH92 α-mannosidase *Bt*3990 from *Bacteroides thetaiotaomicron*, with the substrate-binding site being highly conserved. The function of the CBM32s and other *Nn*GH92 domains was investigated by their sequential deletion and suggested that whilst their binding to the catalytic domain was crucial for the overall structural integrity of the enzyme, they appear to have little impact on the binding affinity to the yeast α-mannan substrate. These new findings provide a better understanding of how to select and optimize other multi-domain bacterial GH92 α-mannosidases for the degradation of yeast α-mannan or mannose-rich glycans.

## Abstract 2

The structure of the full-length multi-domain GH92 α-1,2-mannosidase from *N. novalis* (*Nn*GH92) was solved and the function of the noncatalytic domains was investigated by their sequential deletion and biochemical characterization of the *Nn*GH92 variants. This work significantly advances the structural knowledge of multi-domain GH92 α-1,2-mannosidases and provides a better understanding for the future optimization of these enzymes for the degradation of yeast α-mannan or protein glycans.

## 1. Introduction

**Figure 1:**
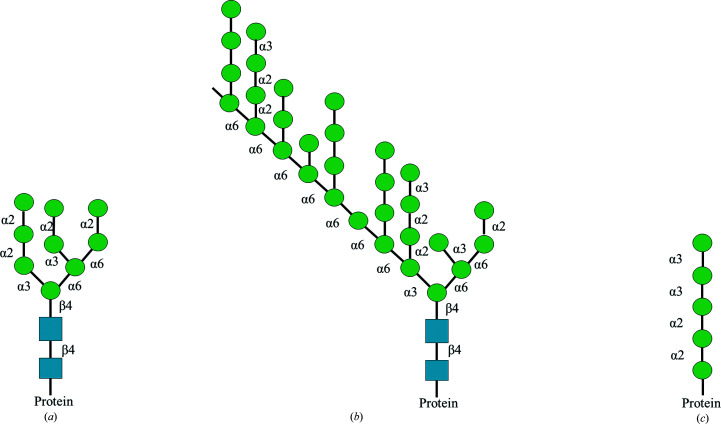
Schematic illustration of the structures targeted by GH92 α-mannosidases. (*a*) The high-mannose N-glycan core (Hakki *et al.*, 2015 ▸). (*b*) α-Mannan found in the outer layer of the yeast cell wall; the α-1,6-linked backbone can reach a degree of polymerization of ∼200 (Orlean, 2012 ▸). (*c*) An O-linked glycan (Goto, 2007 ▸). The proteins produced in *S. cerevisiae* are often decorated with structures (*a*, *c*) that contribute to protein N- and O-glycosylation, respectively. The scheme was inspired by Hakki *et al.* (2015 ▸). The green circles and blue squares are mannose and *N*-acetylglucosamine units, respectively, depicted according to the SNFG guidelines (Varki *et al.*, 2015 ▸; Neelamegham *et al.*, 2019 ▸)

The fungal cell wall consists of polysaccharide layers, including chitin and β-glucans, which provide a scaffold for the mannoproteins present in the outer layer. These glycoproteins are composed of a protein moiety decorated with either O- or N-linked glycans. In *Saccharomyces cerevisiae*, the N-glycans have a high-mannose core backbone (Fig. 1 ▸ *a*) that extends to ∼200 α-1,6-linked mannose units (Fig. 1 ▸ *b*; Abbott *et al.*, 2015 ▸). This α-1,6-linked mannose backbone is decorated with side chains composed of first α-1,2-linked mannose units followed by terminal α-1,3-linked mannose units (Orlean, 2012 ▸).

A key requirement for the complete enzymatic hydrolysis of α-mannan is removal of the side chains that obstruct access to the α-1,6-linked mannan backbone (Cuskin *et al.*, 2015 ▸). α-Mannosidases capable of hydrolysing α-1,2-/α-1,3-glycosidic bonds in the α-mannan side chains belong to the CAZy (Lombard *et al.*, 2014 ▸) glycoside hydrolase family GH92. In 2015, Cuskin and coworkers published a study showing that the human gut bacterium *Bacteroides thetaiota­omicron* had developed a highly specialized enzymatic machinery to degrade yeast α-mannan, releasing short α-oligomannosaccharides and single mannose residues which it can use as a sole carbon source of energy (Cuskin *et al.*, 2015 ▸). This allowed the identification of multiple genes, organized into polysaccharide-utilization loci, encoding enzymes with α-mannosidase or α-mannanase activity. Other α-mannosidases belonging to GH99 and GH38 have been identified which facilitate hydrolysis of the side chains in the degradation of yeast α-mannan by *B. thetaiotaomicron* (Cuskin *et al.*, 2015 ▸; Hakki *et al.*, 2015 ▸).

All known family GH92 members are Ca^2+^-dependent exo-α-mannosidases that perform the hydrolysis of terminal non­reducing mannose residues with inversion of the anomeric configuration (Zhu *et al.*, 2010 ▸). Based on the characterization of the *B. thetaiotaomicron* GH92 α-mannosidases, a wide range of glycosidic bond specificities have been identified, including α-1,2-, α-1,3- and α-1,4-linked mannose linkages (Zhu *et al.*, 2010 ▸). GH92 α-mannosidases were found to play an important role in depolymerization of the *S. cerevisiae* cell-wall α-mannan (Cuskin *et al.*, 2015 ▸) and the mannose-rich N-glycans (Liu *et al.*, 2016 ▸; Li *et al.*, 2020 ▸) or O-glycans (Kołaczkowski *et al.*, 2022 ▸) found in glycoproteins produced by fungi (Fig. 1 ▸).

The structures of several bacterial GH92 α-mannosidases have been reported, including *Bt*3990 (PDB entry 2wvx), *Bt*2199 (PDB entry 2wvy), *Bt*3130 (PDB entry 6f8z) and *Bt*3965 (PDB entry 6f91) from *B. thetaiotaomicron* (Zhu *et al.*, 2010 ▸; Thompson *et al.*, 2018 ▸), *Cc*GH92 from *Cellulosimicrobium cellulans* (PDB entry 2xsg; Tiels *et al.*, 2012 ▸), *Ef*Man-I from *Enterococcus faecalis* (PDB entry 6dwo; Li *et al.*, 2020 ▸) and *Sp*GH92 from *Streptococcus pneumoniae* (PDB entry 5swi; Robb *et al.*, 2017 ▸). All known structures of GH92 α-mannosidase catalytic domains have a highly conserved two-subdomain composition, a N-terminal β-sandwich and a C-terminal (α/α)_6_-barrel, with both subdomains contributing to a pocket-like active site with distinctive −1 and +1 sugar-binding subsites (Zhu *et al.*, 2010 ▸; Davies *et al.*, 1997 ▸). Among these bacterial GH92 structures, a common pattern was identified in the active site, with a highly conserved −1 subsite accommodating the mannosyl nonreducing end and a divergent +1 subsite (Zhu *et al.*, 2010 ▸; Thompson *et al.*, 2018 ▸). The poorly conserved substrate-binding amino-acid residues at the +1 subsite were found to be a structural factor for differentiating the preference of the enzyme towards mannosyl linkages, including α-1,2- (*Bt*3990), α-1,3- (*Bt*3130) and α-1,4-linkages (*Bt*3965) (Thompson *et al.*, 2018 ▸; Zhu *et al.*, 2010 ▸).

In the CAZy database (Lombard *et al.*, 2014 ▸), there are currently 31 ‘characterized’ GH92 α-mannosidases. Among them there is only one enzyme, namely the GH92 αMan2 from *Microbacterium* sp. M-90 (Maruyama *et al.*, 1994 ▸), which possesses an extra domain in addition to the catalytic core domain; this extra domain belongs to carbohydrate-binding module family 32 (CBM32). CBM32 domains display a β-sandwich fold containing a metal ion, usually Ca^2+^, with an exposed shallow-cleft carbohydrate-binding site (Newstead *et al.*, 2005 ▸; Ficko-Blean *et al.*, 2012 ▸; Boraston *et al.*, 2004 ▸). The CBM32s are a family with a wide range of ligand specificities, primarily targeting nonreducing ends of complex glycans such as the mucin type (Ficko-Blean & Boraston, 2006 ▸), including galactose, lactose (Newstead *et al.*, 2005 ▸) and *N*-acetyllactos­amine (LacNAc; Boraston *et al.*, 2007 ▸). Recently, several CBM32s have been identified appended to GH enzymes catalysing the degradation of plant cell-wall polysaccharides, including pectin (Lyu *et al.*, 2018 ▸) and β-mannan (Mizutani *et al.*, 2012 ▸). No CBM32 was present in any of the 22 GH92 α-mannosidases from *B. thetaiotaomicron* and the binding specificity of the CBM32s was not assigned to α-mannan or other glycan-containing α-mannooligosaccharides.

Here, we report the biochemical characterization and the crystal structure of the full-length multi-domain GH92 α-mannosidase from *Neobacillus novalis* (*Nn*GH92), a bacterium identified in agricultural soils (Heyrman *et al.*, 2004 ▸; Patel & Gupta, 2020 ▸). *Nn*GH92 is additionally important as it has recently been used in an enzymatic technique to map fungal high-mannose structures (Kołaczkowski *et al.*, 2022 ▸). The 3D structure was solved with the known GH92 inhibitor mannoimidazole (ManI) bound in the active site. Sequence and structural alignments were performed with the known GH92 α-1,2-mannosidase *Bt*3990 from *B. thetaiotaomicron* to identify the general acid (Ly & Withers, 1999 ▸), the Brønsted base (Ly & Withers, 1999 ▸) and the ligand interactions in the Ca^2+^-containing active site. Domain-deletion variants were designed and expressed to evaluate the influence of each noncatalytic domain on the activity and binding ability of the enzyme to yeast α-mannan and the isolated yeast cell wall from *S. cerevisiae*.

## 2. Methods

### 2.1. Materials

α-1,2-Mannobiose, α-1,3-mannobiose and α-1,6-mannobiose were purchased from Dextra. Unless stated otherwise, all other chemicals were purchased from Sigma–Aldrich. α-Mannan from the *S. cerevisiae mnn2* mutant (α-1,6-linked mannan backbone without side chains) was extracted as described previously (Raschke *et al.*, 1973 ▸). The same extraction was performed for wild-type *S. cerevisiae* for use as a control α-mannan.

### 2.2. Cloning, expression and purification of wild-type *Nn*GH92 and its variants

The data for the GH92 α-1,2-mannosidase from *N. novalis* (*Nn*GH92) were deposited in the European Nucleotide Archive (ENA) at EMBL–EBI with accession No. LR963497.1 (GenBank sequence ID). The design of the variants was based on the structure of *Nn*GH92, targeting the linker regions between the catalytic domain and the associated noncatalytic domains or between both associated noncatalytic domains. The following deletions were introduced into the variants, where the numbers correspond to the deleted range of amino-acid residues from the wild type: ΔFHB, Δ1319–1411; ΔFHBCBM32, Δ1168–1411; ΔN-CBM, Δ34–397; core, Δ34–397 and Δ1168–1411. All constructs were verified by sequencing.

The wild type and the variants were cloned and expressed as extracellular enzymes in *Bacillus subtilis* in a similar setup as described previously (Jensen *et al.*, 2010 ▸) with the following modifications. The native signal peptide was replaced by the Alcalase signal peptide followed by a histidine tag (6×His + PR), resulting in the N-terminal sequence MKKPLGKIVASTALLISVAFSSSIASAHHHHHHPR. The fermentation broth was sterile filtrated, and 500 m*M* NaCl was then added and adjusted to pH 7.5 with NaOH. The sample was loaded onto an Ni Sepharose 6 Fast Flow column (GE Healthcare, Piscataway, New Jersey, USA) equilibrated in 50 m*M* HEPES pH 7.5 with 500 m*M* NaCl (buffer *A*). After loading, the column was washed with ten column volumes of buffer *A* and the bound proteins were eluted with 500 m*M* imidazole in buffer *A*. The fractions containing the enzyme were pooled and applied onto a Sephadex G-25 (medium) column (GE Healthcare, Piscataway, New Jersey, USA) equilibrated and eluted with 50 m*M* HEPES pH 7.5. Fractions were analysed by SDS–PAGE and those containing the enzyme were combined. Protein concentrations were determined by measuring the absorption at 280 nm with a NanoDrop 8000 spectrophoto­meter (Thermo Scientific) using extinction coefficients based on the amino-acid sequences of the *Nn*GH92 enzymes. The identity of the purified enzymes was verified by excising the protein bands from the SDS–PAGE gel (Supplementary Fig. S1) and analysing a tryptic digest by mass spectrometry.

### 2.3. Enzyme-assay conditions

All enzyme-activity assays were conducted in an assay buffer composed of 50 m*M* MES pH 6.0, 50 m*M* NaCl, 2 m*M* CaCl_2_, 0.01% Triton X-100 unless stated otherwise. Enzymatic hydrolysates were quenched with 0.15 *M* NaOH and analysed with a reducing-sugar assay (PAHBAH) to quantify the reducing-sugar ends (Lever, 1973 ▸) or high-performance anion-exchange chromatography/pulsed amperometric detection (HPAEC-PAD) to quantify the released mannose concentration. The detailed experimental procedure has been described elsewhere (Sørensen *et al.*, 2015 ▸). The absorption of the coloured products was measured at 405 nm using a plate reader (SpectraMax 3; Molecular Devices). The absorbance readouts were recalculated to the concentration of reducing ends using a mannose standard curve (0–5 m*M*). The substrate conversion was calculated as the actual yield/theoretical yield × 100%. The hydrolysis of yeast α-mannan by *Nn*GH92 resulted in products with a degree of polymerization (DP) of 1 and only mannose was identified by HPAEC-PAD. Thus, the measured concentration of sugar reducing ends was assumed to be equal to the mannose reducing ends (actual yield). The yeast α-mannan was acid hydrolysed, the released monosaccharides were quantified by HPAEC-PAD as described elsewhere (Schiano-di-Cola *et al.*, 2020 ▸) and the mannose concentration was calculated. This concentration was divided by the initial yeast α-mannan concentration corrected for the monomeric units (180/162) to obtain the theoretical yield. The sample analyses using HPAEC-PAD followed the procedure described previously (Schiano-di-Cola *et al.*, 2020 ▸).

### 2.4. Thermal stability

The studied enzymes were analysed by nano differential scanning fluorimetry (nanoDSF; Prometheus NT.48, NanoTemper) to determine the melting temperature (*T*_m_). The enzymes were diluted to a concentration of 2 mg ml^−1^ in 50 m*M* MES pH 6.0. The thermal stability was tested with a heating scan range from 20 to 90°C at a scan rate of 2°C min^−1^. Data analysis and calculation of *T*_m_ were performed using the *PR.ThermControl* software (NanoTemper).

### 2.5. pH and temperature optima

The pH profile was calculated by enzymatic hydrolysis of 5 mg ml^−1^ α-mannan solubilized in assay buffers with different buffer components: 50 m*M* sodium acetate pH 3.6, 4.0 or 5.0, 50 m*M* MES pH 6.0, 50 m*M* HEPES pH 7.0 or 8.0 or Tris pH 9.0 with 13 n*M* *Nn*GH92 with an incubation time of 2 h at 25°C. Enzymatic hydrolysates were withdrawn at different time points (15, 30, 45, 75 and 120 min) and analysed by the reducing-sugar assay (PAHBAH). Based on the absorbance measurement at 405 nm, the linear range of the reaction progress curve was calculated; the highest activity was set to 1 and the rest was normalized with the same factor.

The temperature profile was calculated at five different points (20, 33, 42, 52 and 60°C) using the assay buffer at pH 6. The other calculations were performed following the same procedure as for the pH profile.

### 2.6. Kinetics of wild-type *Nn*GH92 with α-mannobiose

The kinetic constants (*k*_cat_ and *K*_m_) of wild-type *Nn*GH92 were determined by assaying the initial hydrolysis rate at 37°C using different α-1,2-mannobiose concentrations. The release of mannose was quantified using the Megazyme International kit for d-mannose assay (K-MANGL, Megazyme) and a mannose standard curve. The corresponding substrate concentrations were prepared by dissolving α-1,2-mannobiose in assay buffer (see Section 2.3). The enzyme concentration used for the assay was 7 n*M*. The initial rates were plotted as a function of α-1,2-mannobiose concentration and were fitted with the Michaelis–Menten equation. A similar experiment was attempted for α-1,3-mannobiose except that 66 n*M* wild-type *Nn*GH92 was used; however, it was not possible to fit the Michaelis–Menten equation due to insufficient initial rate points.

### 2.7. Thin-layer chromatography (TLC)

A few droplets (3–4 µl) of the completed reaction mixture of hydrolysis by *Nn*GH92 were spotted onto silica-gel TLC plates (stationary phase). Once the sample spots had dried, the plates were immersed in a solution of butanol:acetic acid:water mixed in a 2:1:1 ratio (mobile phase). The plate was developed until the mobile phase reached 80–90% of the full height of the plate. The plate with the separated components was dried with a hot-air gun and carbohydrates were detected by immersing the plate in chemical stain (5% ammonium molybdate, 0.02% cerium sulfate, 5% sulfuric acid). After a few seconds, the plate was removed and dried again until the blue bands developed (Cuskin *et al.*, 2015 ▸). Standards were prepared by solubilizing 1 mg ml^−1^ β-mannooligosaccharides (Megazyme) with a DP of 2–6 and 1 mg ml^−1^ mannose (Sigma). The DP of the sample was estimated by comparing the bands for the sample with the lane containing sugar standards.

### 2.8. Crystallization

Crystallization experiments were carried out in the presence or absence of 8 m*M* CaCl_2_ and 5 m*M* mannoimidazole. Hits were only obtained for the mannoimidazole complex with CaCl_2_ in PACT premier HT-96 (Molecular Dimensions) conditions B7 (0.2 *M* NaCl, 0.1 *M* MES pH 6.0, 20% PEG 6K) and E6 (0.2 *M* sodium formate, 20% PEG 3350). The crystals were imperfect and were used to make seed stock. The seed stock was prepared and microseed matrix screening (MMS; for a review, see D’Arcy *et al.*, 2014 ▸) was carried out using an Oryx robot (Douglas Instruments) according to published protocols (Shah *et al.*, 2005 ▸; Shaw Stewart *et al.*, 2011 ▸). Briefly, crystals were crushed and diluted with ∼50 µl mother liquor. The solution was transferred into a reaction tube containing seed beads and vortexed for 3 min. The seed stock was used immediately and any remaining seeds were frozen and kept at −20°C. MMS was carried out in the PACT screen, giving an increased number of improved hits. Crystals from condition F11 (0.2 *M* sodium citrate, 0.1 *M* bis-Tris propane pH 6.5, 20% PEG 3350) were used to make a seed stock for the next seeding rounds into optimization screens based on the successful conditions with different seed dilutions. The crystallization drops consisted of 150 nl protein (including 8 m*M* CaCl_2_ and 5 m*M* mannoimidazole), 50 nl seed stock and 100 nl mother liquor from a new random screen. The final crystal was obtained in 21% PEG 3350, 0.1 *M* bis-Tris propane pH 6.6, 0.2 *M* sodium citrate.

### 2.9. Data collection, structure solution and refinement

All computation was carried out using programs from the *CCP*4 suite (Winn *et al.*, 2011 ▸) unless stated otherwise. Data were collected to 2.3 Å resolution on beamline I04-1 at Diamond Light Source, integrated using *XDS* (Kabsch, 2010 ▸) within the *xia*2 pipeline (Winter *et al.*, 2013 ▸) and scaled with *AIMLESS* (Evans & Murshudov, 2013 ▸). The space group was *P*2_1_, with unit-cell parameters *a* = 94.61, *b* = 151.940, *c* = 114.01 Å, β = 94.63°. The structure was solved by molecular replacement with *MOLREP* (Vagin & Teplyakov, 2010 ▸) using PDB entry 2wzs (the family GH92 inverting mannosidase *Bt*3990 from *B. thetaiotaomicron* VPI-5482 in complex with mannoimidazole; Zhu *et al.*, 2010 ▸) as a model. 60 cycles of jelly-body refinement with *REFMAC* (Murshudov *et al.*, 2011 ▸) were followed by density modification with *Parrot* (Cowtan, 2010 ▸) and the initial model was built with *Buccaneer* (Cowtan, 2006 ▸). Further refinement was carried out in *REFMAC* with the TLS option iterated alternated with manual model correction in *Coot* (Emsley *et al.*, 2010 ▸). The quality of the final model was validated using *MolProbity* (Chen *et al.*, 2010 ▸) as part of the *Phenix* package(Adams *et al.*, 2011 ▸). The final data-processing and refinement statistics are given in Table 1 ▸.

### 2.10. Yeast cell-wall extraction

Yeast cell walls were extracted from *S. cerevisiae* cells grown in sterile YPD medium for three days at 32°C and 150 rev min^−1^. The yeast cells were harvested and washed three times with cold deionized water by centrifugation at 4000 rev min^−1^ for 10 min. Yeast cells were diluted in 10 m*M* Tris–HCl pH 8.0 at a concentration of 50 mg cell wet mass per millilitre. Extraction of yeast cell walls was conducted with a cell disruptor (CF1 model, Constant Systems) at a pressure of 124 MPa. Four passages were applied to ensure complete disruption of the yeast cells. Subsequently, the extracted cell walls were pelleted at 3800*g* for 5 min, washed with cold water until the supernatant became clear and stored at 4°C (Dallies *et al.*, 1998 ▸). The yeast cell walls were then washed three times and resuspended in 50 m*M* MES pH 6.0, 50 m*M* NaCl, 2 m*M* CaCl_2_. The final concentration of the yeast cell-wall stock was calculated as a dry cell weight and it was used in the activity and binding assays.

### 2.11. Native affinity gel electrophoresis

The ability of *Nn*GH92 variants to bind to a soluble yeast α-mannan was evaluated by native affinity gel electrophoresis. The materials, assay and data analysis were performed according to the protocol demonstrated elsewhere (Cockburn *et al.*, 2017 ▸) with the following changes: the gel was composed of 10% acrylamide and 0.1% yeast α-mannan in 50 m*M* Tris pH 8.7. Each lane was loaded with 4 µg enzyme. Both a control and polysaccharide gels were run in 50 m*M* Tris pH 8.7 for 20 h at 4°C at a constant 75 V. In the control gel, the yeast α-mannan was substituted with 50 m*M* Tris pH 8.7. The gels were prepared without the stacking layer.

### 2.12. Yeast cell-wall binding and activity assay

*Nn*GH92 variants at different enzyme concentrations were mixed with 20 g l^−1^ (dry cell weight, DWC) insoluble yeast cell-wall extract from *S. cerevisiae* solubilized in assay buffer omitting 0.01% Triton X-100 and equilibrated for 1 h at 4°C and 1110 rev min^−1^. The resulting mixtures were centrifuged (16 800 rev min^−1^) at 4°C and the amounts of unbound protein were obtained using a spectrophotometric method measuring the absorbance at 280 nm. The calculation of free enzyme and bound enzyme and fitting using the Langmuir isotherm was performed as described elsewhere (Kołaczkowski *et al.*, 2020 ▸). Activity assays of *Nn*GH92 variants were performed on the same substrate. The enzymes at two enzyme concentrations, 0.1 and 1 µ*M*, were mixed with 60 g l^−1^ DWC extracted yeast cell wall solubilized in assay buffer and incubated at 37°C for 1 h. The hydrolysates were analysed with the reducing-sugar assay (PAHBAH) and the absorbance measurements were recalculated to give the mannose concentration using a mannose standard curve.

## 3. Results

The full-length sequence of *Nn*GH92 was deposited in GenBank with code LR963497.1. In contrast to *B. thetaiota­omicron*, which produces GH92 α-mannosidases as solely catalytic domains, *Nn*GH92 has five domains, as discussed below. A gene encoding full-length *Nn*GH92 was cloned and expressed in *B. subtilis*. The construct used for the structural study excludes the signal peptide and therefore started from Ser34 with an N-terminal His tag (HHHHHHPR). After purification, it showed a single band on SDS–PAGE analysis (Supplementary Fig. S1, lane 2).

### 3.1. Overall structure of *Nn*GH92

**Figure 2:**
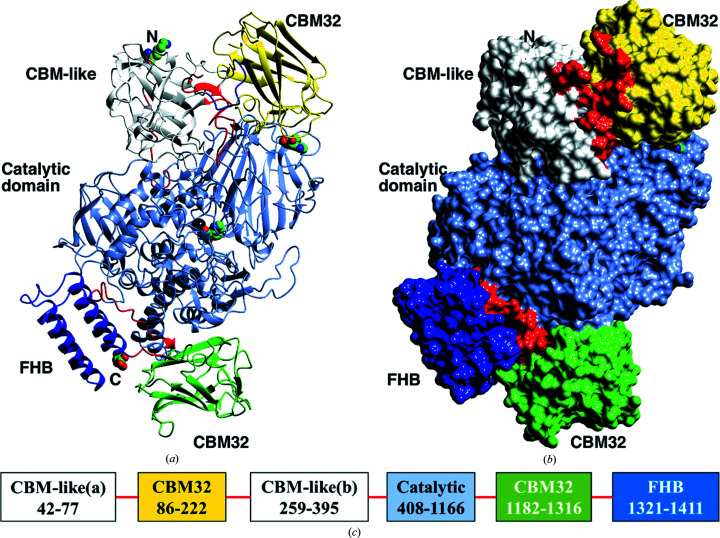
The structure of *Nn*GH92 (PDB entry 7nsn). (*a*) The fold of chain *A* in ribbon format. The domains are coloured from the N-terminus: CBM-like (white; the fold is split by the first CBM32 insert), CBM32 (lemon), the catalytic domain (ice blue), the second CBM32 (green) and the four-helix bundle (FHB; blue). The linkers between the domains are shown in red and are ordered in the structure. Both mannoimidazoles (ManI) are shown as spheres coloured by atom type: the first at the active site between the two subdomains of the catalytic domain and the second between the catalytic domain and the N-­terminal CBM32. The calcium ion adjacent to the active-site ManI is shown as a black sphere. The N-terminal residue (Lys42) and C-terminal residue (Asp1411) are shown as spheres. (*b*) The surface of the domains coloured as in (*a*). The extensive packing surfaces of the domains is evident. The ordered linkers between domains are highlighted in red. The images were created with *CCP*4*mg* (McNicholas *et al.*, 2011 ▸). (*c*) Schematic representation of the domain structure of *Nn*GH92. CBM-like(a) and CBM-like(b) correspond to the same domain structure.

With the aim of confirming how *Nn*GH92 accommodates and interacts with mannopyranosides, and to establish the location and possible roles of the associated noncatalytic domains, the crystal structure of the wild type was solved in complex with the mannosidase inhibitor mannoimidazole (ManI; PDB entry 7nsn; Fig. 2 ▸). The structure was solved by molecular replacement using the published structure of *Bt*3990 (PDB entry 2wzs; Zhu *et al.*, 2010 ▸) as a template and was refined at 2.3 Å resolution (Table 1 ▸). *Nn*GH92 only crystallized in the presence of ManI, suggesting that the ligand binding improved the structure stability, leading to the formation of high-quality crystals. Most importantly, the structure corresponded to the full-length enzyme including all of the noncatalytic domains (Fig. 2 ▸), at least in chain *A*.

There are two independent monomers in the asymmetric unit. Chain *A* consists of residues 42–1411. A few residues at the N-terminus, including the His tag, are disordered with no electron density. The rest of chain *A* is well ordered in the crystal, with the exception of a short loop of five residues, 224–227. At the N-terminus there is the start of an all-β-sheet domain reminiscent of a CBM and termed ‘CBM-like’, which is made up of residues 42–77 and 259–395. A CBM32 (residues 86–222) is inserted into a loop of the CBM-like domain. These are followed by the catalytic domain (residues 408–1166), the C-terminal CBM32 (residues 1182–1316) and a four-helix bundle domain (FHB; residues 1321–1411). In chain *B*, there were only very poor fragments of density for the N-terminal CBM32 domain, and residues 79–227 are missing from the model. The rest of the fold of the *A* and *B* chains is essentially identical, with an r.m.s.d. of 0.27 Å over 1221 equivalent C^α^ atoms. This supports a stable set of interactions for the extra domains surrounding the core catalytic domain. The rest of the description will focus on the better ordered chain *A*. The catalytic domain was traced without any breaks and its fold, as expected, was very similar to that of *Bt*3990, with an r.m.s.d. of 2.01 Å over 712 equivalent C^α^ atoms reflecting the moderate sequence identity (41.6%). The chain of the catalytic domain adopted the expected two-subdomain structure: an N-terminal β-sandwich and a C-terminal (α/α)_6_-barrel (Fig. 2 ▸ *a*). Superposition of the *Bt*3990 and *Nn*GH92 crystal structures showed no difference in structural elements for the catalytic domains. The active site is a shallow pocket, with both the N- and C-terminal domains contributing to its shape.

**Figure 3:**
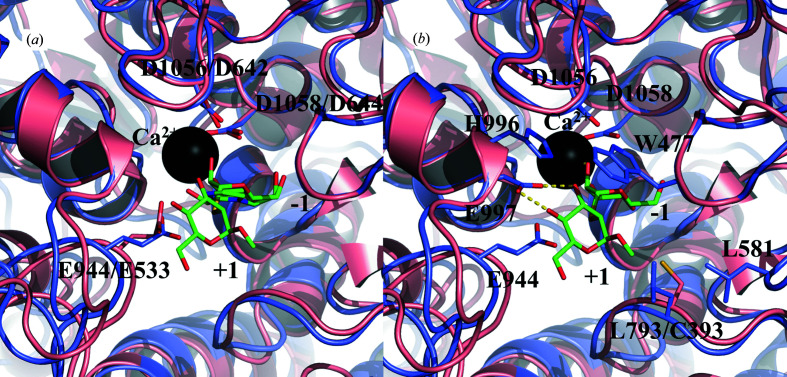
Comparison of the active sites of *Nn*GH92 (blue) and *Bt*3990 (pink) with α-mannosidase inhibitors. The *Bt*3990–MSM complex (PDB entry 2ww3) was superimposed on the *Nn*GH92–ManI complex using *PyMOL* and its built-in *cealign* function. The calcium ion is coloured black. (*a*) The general acid (Glu944/533) and two general bases Asp1056/642 and Asp1058/644 align at equivalent positions. At the −1 subsite, the mannose ring of ManI aligns with the nonreducing end of MSM. (*b*) Amino-acid residues shaping the binding subsites and interacting with the ligands. At the +1 subsite, Leu793 in *Nn*GH92 was in an equivalent position to Cys393 in *Bt*3990, whereas the other residues were highly conserved in similar orientations. The structures were visualized using *PyMOL* (version 2.3.2; Schrödinger).

The reaction mechanism of GH92 enzymes, with catalysis occurring with inversion of the anomeric configuration, requires two residues: an acid to assist the departure of the leaving group and a base to enhance the nucleophilic attack of water (Zhu *et al.*, 2010 ▸). The catalytic residues, the Brønsted acid Glu944 (Glu533 in *Bt*3990) and the Brønsted base Asp1058 (Asp644 in *Bt*3990), are conserved (Fig. 3 ▸ *a*, Supplementary Fig. S2; Ly & Withers, 1999 ▸). To further investigate the local features in the active site, the structure of *Nn*GH92 was superimposed on that of *Bt*3990 in complex with thio-linked α-1,2-mannobiose (MSM; PDB entry 2ww3; Zhu *et al.*, 2010 ▸; Fig. 3 ▸). Like *Bt*3990, *Nn*GH92 aligns MSM with a clear boundary between the −1 and +1 subsites indicated by the nonhydrolysable glycosidic S atom. Moreover, the ManI mannose ring superimposes on the mannose ring of MSM at the −1 subsite. The residues providing interactions in both subsites are highly conserved, with a nearly identical orientation and position in both enzymes (Fig. 3 ▸ *b*). At the −1 subsite, the Ca^2+^ ion supports the positioning of the sugar ring by interacting with O2 and O3 of the mannose. The specificity of the α-1,2-mannosidase *Bt*3990 is primarily driven by three residues at the +1 subsite: the His584-Glu585 pair of residues forming hydrogen bonds to O3 and O4 of the MSM mannose and Trp88 providing a hydrophobic interaction (Thompson *et al.*, 2018 ▸). Equivalent residues (His996-Glu997 and Trp477) are present in the +1 subsite of *Nn*GH92 (Fig. 3 ▸ *b*, Supplementary Fig. S2). These structural findings, together with the observed activity on α-1,2-mannobiose (Kołaczkowski *et al.*, 2022 ▸; Supplementary Figs. S3 and S4), confirmed the assignment of *Nn*GH92 as an α-1,2-mannosidase. Interestingly, two residues, Leu581 and Leu793, enter the +1 subsite from the right (Fig. 3 ▸ *b*). In the structures of *Bt*3990, *Bt*2199 and *Bt*3130, Cys399 is present instead of Leu793 in *Nn*GH92 (Fig. 3 ▸ *b*). Despite Cys399 not having been identified as being involved in any interaction with the sugar moiety at the +1 subsite, the hydrophobic nature of the two leucine residues may be involved in coordinating other extended glycan chains such as yeast α-mannan side chains. The absence of the residues conferring α-1,3-mannosidase specificity (identified in *Bt*3130; Thompson *et al.*, 2018 ▸) in the +1 subsite of *Nn*GH92 probably explains its low activity towards α-1,3-mannobiose (Kołaczkowski *et al.*, 2022 ▸).

### 3.2. The structure of the *Nn*GH92 noncatalytic domains

**Figure 4:**
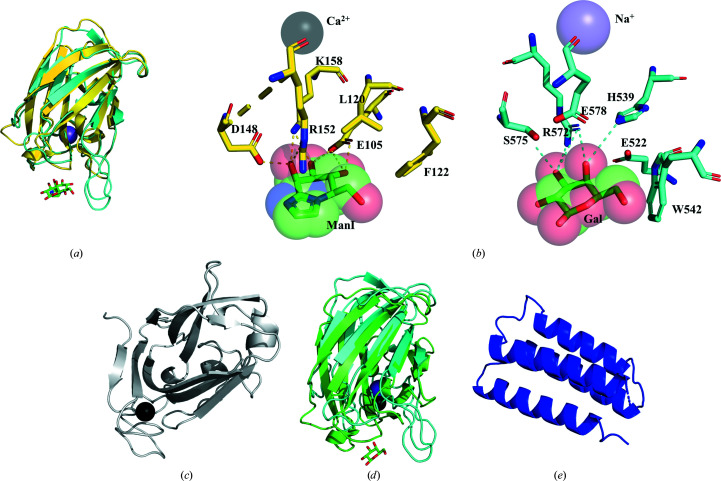
The structure of the noncatalytic domains of *Nn*GH92. (*a*) The N-terminal CBM32 (yellow) superimposed on the CBM32 of the GH33 sialidase from *Micromonospora viridifaciens* (*Mv*GH33; cyan; PDB entry 1euu; Gaskell *et al.*, 1995 ▸). (*b*) ManI (coloured by atom type) bound to the N-terminal CBM32 (yellow) and galactose (Gal, coloured by atom type) bound to the CBM32 (cyan) of *Mv*GH33. The highlighted residues shape the binding sites of both CBM32s. The Na^+^ ion (purple) is in an equivalent position to the Ca^2+^ atom (black) in *Nn*GH92. (*c*) The CBM-like domain (grey) with a bound Ca^2+^ atom (black). (*d*) The C-terminal CBM32 (green) superimposed on the CBM32 of *Mv*GH33 (cyan; PDB entry 1euu; Gaskell *et al.*, 1995 ▸). (*e*) The FHB domain (blue) of unknown function.

The structure of *Nn*GH92 contained several domains in addition to the catalytic domain (Figs. 2 ▸ and 4 ▸). Both CBM32s had the β-sandwich fold architecture typical of this family, with five- and three-stranded antiparallel β-sheets opposing one another (Figs. 4 ▸ *a* and 4 ▸ *d*). Ca^2+^ ions were buried within the structures of these CBM32s, a common feature of the CBM32 family (Ficko-Blean & Boraston, 2009 ▸; Ficko-Blean *et al.*, 2012 ▸; Boraston *et al.*, 2007 ▸; Abbott *et al.*, 2008 ▸).

There was clear electron density for a single ManI bound to the N-terminal CBM32. A CBM32–galactose complex from *Micromonospora viridifaciens* GH33 sialidase (*Mv*GH33; PDB entry 1euu; Gaskell *et al.*, 1995 ▸) was overlaid on this domain with an r.m.s.d. of 2.29 Å over 128 equivalent C^α^ atoms and a sequence identity of 31.1% (Fig. 4 ▸ *a*). In the overlaid structures of *Nn*GH92 CBM32 and *Mv*GH33 CBM32 (*Mv*CBM32), both ligands (ManI and galactose) and the metal ions (Ca^2+^ and Na^+^) lie in equivalent positions, confirming the ligand-binding site of the N-terminal CBM32 of *Nn*GH92. In Fig. 4 ▸(*a*), the major difference is visible in the loop covering the C6 group of the galactose and ManI, where *Mv*CBM32 is more extended with Trp542 (Fig. 4 ▸ *b*). While the loop is much shorter in the N-terminal CBM32, it has an aromatic residue, Phe122 (Fig. 4 ▸ *b*), with a similar orientation, providing stacking interactions with the C6 group of ManI. In *Mv*CBM32, Arg572 and Glu578 form hydrogen bonds to O3/O4 and O4 of the galactose ring, respectively. In the N-terminal CBM32, Arg152 has a similar orientation to Arg572 but is more distantly located, only forming a hydrogen bond with O2 of ManI. In addition, Asp148 O2 and Glu105 O3/O4 form hydrogen bonds to the mannose ring of ManI. The two ManI ligands at the active site and the binding site of the N-terminal CBM32 are ∼35 Å from one another and lie along the same axial plane, probably to facilitate the movement of the N-terminal CBM32 and the catalytic domain (CD) to bring the substrate closer to the active site.

Following the N-terminal CBM32, there is another domain, which despite its β-sheet and similar ‘CBM-like’ architecture including Ca^2+^ cannot be assigned to any known CBM family based on sequence alignment (Fig. 4 ▸ *c*). Structural alignment with *Gesamt* (Krissinel & Uski, 2017 ▸) led to the identification of the closest homologue (PDB entry 1pmh; r.m.s.d. of 2.9 Å over 157 equivalent C^α^ atoms, *Q*-score 0.34), which belongs to the CBM27–mannohexaose complex associated with a GH26 β-mannanase (Roske *et al.*, 2004 ▸). This CBM27 interacts with β-mannooligosaccharides through an aromatic platform formed by tryptophan residues (Trp23, Trp60 and Trp113). Despite having Ca^2+^ atoms at equivalent positions, the aromatic platform is absent in the *Nn*GH92 CBM-like domain. Based on the very low sequence identity (7%) between CBM27 and the CBM-like domain, and the absence of bound ligand, it was not possible to assign a carbohydrate-binding site for this domain. Therefore, the CBM-like domain could either have a purely structural function or could function to block the access of unfavourable glycan chains to the enzyme active site, prioritizing only shorter glycan chains. A sequence homologue of the ‘CBM-like’ domain is also found in family GH38 α-mannosidases from *Clostridium* spp. (BCI61027.1 and BCI60986.1).

While the C-terminal CBM32 has a high structural similarity to the N-terminal CBM32 (r.m.s.d. of 1.9 Å over 128 equivalent C^α^ atoms), they share only limited sequence identity (36%). The C-terminal CBM32 was also superimposed on *Mv*CBM32 to indicate a potential binding site (Fig. 4 ▸ *d*). Similar to the N-terminal CBM32, the C-terminal CBM32 putative binding site is found in the same axial plane, with a distance of ∼40 Å to the active site of *Nn*GH92.

The FHB domain is composed of a bundle of four α-helices (Fig. 4 ▸ *e*) oriented towards the exterior of the *Nn*GH92 structure (Fig. 2 ▸). Its closest structural homologue is the bacterial protein EntA from *Enterococcus faecium* (PDB entry 2bl8; r.m.s.d. of 2.8 Å over 72 equivalent C^α^ atoms), which belongs to a group of immunity proteins conferring protection of bacteriocin-producing organisms against their own bacteriocins (Johnsen *et al.*, 2005 ▸). Due to the very low sequence identity (∼6%), it is not possible to assign any function to the FHB domain. Other close structural homologues with low sequence identity (below 10%) were identified, but no functions were provided for these domains (PDB entries 2qzg, 2qsb and 2rld). A three α-helix bundle is also appended at the C-terminus, in series with CBM32s, to a GH84 β-*N*-acetyl­glucosaminidase from *Clostridium perfringens* (Ficko-Blean *et al.*, 2009 ▸).

### 3.3. Design of *Nn*GH92 variants and biochemical characterization

**Figure 5:**
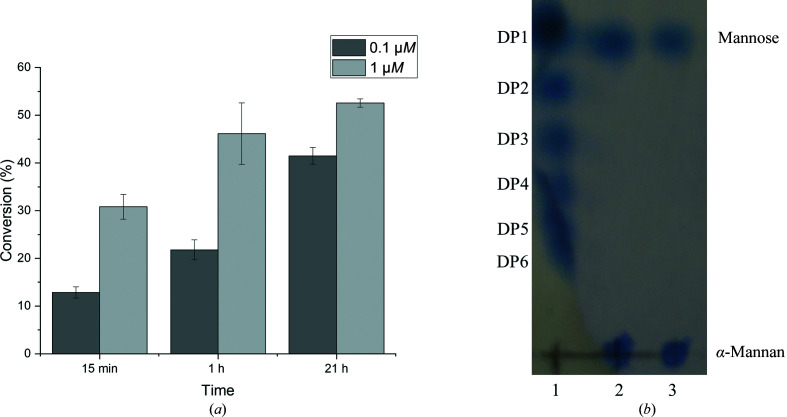
Yeast α-mannan hydrolysis by *Nn*GH92. (*a*) The hydrolysates were analysed at three different time points using the reducing-sugar assay (PAHBAH). The degree of conversion was calculated based on the total mannose released after strong acid hydrolysis (see Section 2). (*b*) Product release pattern analysed by TLC. Lane 1 represents standards composed of β-1,4-mannooligosaccharides with a degree of polymerization (DP) between 1 and 6. Lanes 2 and 3 correspond to the α-mannan hydrolysates after 1 h at enzyme concentrations of 0.1 and 1 µ*M*. Only a single band was observed, corresponding to DP1.

As previously demonstrated, among the tested α-1,2-, α-1,3- and α-1,6-linked mannobioses, *Nn*GH92 exhibited a preference towards α-1,2-mannobiose, confirming that it is α-1,2-mannosidase (Kołaczkowski *et al.*, 2022 ▸). Upon longer incubation (19 h) α-1,3-mannobiose was also partially hydrolysed, while no activity was detected on α-1,6-mannobiose (Kołaczkowski *et al.*, 2022 ▸). *Nn*GH92 had a pH optimum between 6 and 7 and a temperature optimum between 42 and 52°C. The thermal stability, measured as the melting temperature (*T*_m_), was found to be 60°C in the presence of Ca^2+^ at pH 6 (Table 2 ▸). As expected, *Nn*GH92 was active on yeast α-mannan, reaching a degree of conversion of approximately 50% after 1 h (Fig. 5 ▸ *a*). This suggests that it is likely to hydrolyse both the α-1,3- and α-1,2-glycosidic bonds present in the α-mannan side chains, as previously demonstrated for other GH92 α-mannosidases (Maruyama *et al.*, 1994 ▸; Cuskin *et al.*, 2015 ▸; Zhu *et al.*, 2010 ▸). Furthermore, *Nn*GH92 did not exhibit activity on α-mannan from the *S. cerevisiae* yeast *mnn2* mutant (Raschke *et al.*, 1973 ▸) comprised of only α-1,6-linked mannan backbone without side chains. Based on the high degree of conversion of yeast α-mannan by *Nn*GH92 (Fig. 5 ▸ *a*), the enzyme was tested in the presence of GH76 endo-α-1,6-mannanase and GH125 exo-α-1,6-mannosidase to investigate whether the combination of all three enzymes could boost α-mannan degradation. Indeed, all three enzymes improved the degree of conversion, indicating complete depolymerization of yeast α-mannan (Supplementary Fig. S5). Thin-layer chromatography (TLC) was used to provide a qualitative profile of the products generated by *Nn*GH92 on yeast α-mannan. After 1 h of hydrolysis, only a single band was observed on the TLC plate. This confirms that *Nn*GH92 releases monosaccharide as its main enzymatic reaction product (Fig. 5 ▸ *b*), consistent with the exoglycosidase action of GH92 enzymes (Cuskin *et al.*, 2015 ▸; Zhu *et al.*, 2010 ▸).

The Michaelis–Menten kinetic parameters with α-1,2-mannobiose were determined (Supplementary Fig. S3) and compared with those of the model GH92 α-1,2-mannosidase *Bt*3990 (Zhu *et al.*, 2010 ▸). The maximum turnover (*k*_cat_) of *Nn*GH92 was found to be approximately three times higher (13.6 × 10^3^ ± 3.7 × 10^3^ min^−1^) compared with *Bt*3990 (5.2 × 10^3^ ± 3 × 10^2^ min^−1^) (Zhu *et al.*, 2010 ▸). Thus, the presence of the associated domains in *Nn*GH92 appears to have an impact on the overall rate of hydrolysis. In addition, the Michaelis constant (*K*_m_) was calculated, indicating a slightly lower substrate affinity of *Nn*GH92 (0.46 ± 0.22 m*M*) than *Bt*3990 (0.76 ± 0.11 m*M*) (Zhu *et al.*, 2010 ▸).

To elucidate the biochemical role of the associated domains in *Nn*GH92, a number of N- and C-terminally truncated variants were designed (Table 2 ▸). Deletions of the selected domains were designed based on the full-length structure. The inactive variant was created by mutating the general acid Glu944 to Asp, which has previously been demonstrated to suppress the activity of *Bt*3990 (Zhu *et al.*, 2010 ▸). The variants were successfully expressed and purified. Based on SDS–PAGE analysis (Supplementary Fig. S1), the deletions affected the structural integrity of *Nn*GH92 to varying degrees. For the variants with the C-terminal deletions (ΔFHB and ΔFHBCBM32) the top band corresponded to the molecular weight of the enzyme variants followed by two additional bands with a lower molecular weight (Supplementary Fig. S1, lanes 3–4). The N-terminal deletions (ΔN-CBM and core variants) had a much more severe impact, showing only a very low-intensity band corresponding to the full-length variant followed by multiple bands corresponding to proteins with different molecular weights (Supplementary Fig. S1, lanes 5–6). In-gel digestion of the selected bands from the SDS–PAGE (Supplementary Fig. S1) followed by mass-spectrometric analysis indicated that all of the protein bands had the expected molecular weight calculated for that variant (data not shown).

**Figure 6:**
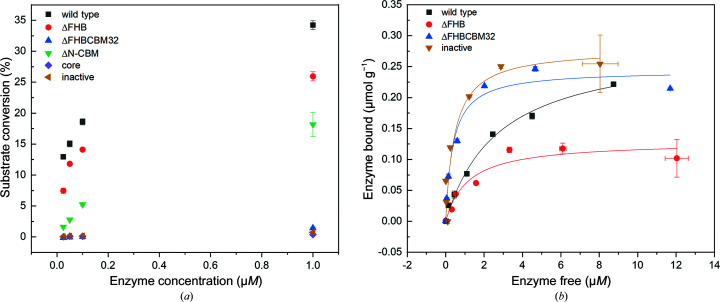
Activity and binding profiles of the *Nn*GH92 variants. (*a*) Yeast α-mannan hydrolysis. The variants at different enzyme concentrations were mixed with 2.5 g l^−1^ yeast α-mannan and incubated for 1 h at 37°C. The activity was calculated based on the release of the reducing sugar ends (PAHBAH). (*b*) The variants binding to yeast cell-wall extracts (see Section 2). No binding was found for the ΔN-CBM and core variants and control (BSA). Solid lines represent the fitted Langmuir equation. Error bars represent standard deviations from triplicate measurements.

The *T*_m_ of the variants was significantly lower, by ∼10°C, than that of the wild-type and inactive variants (Table 2 ▸). As expected, the activity of the variants was also impacted by the absence of the associated domains (Fig. 6 ▸ *a*). The activity on the yeast α-mannan was least impaired for the ΔFHB variant, reaching approximately 80% of the wild-type activity at 1 µ*M* enzyme concentration. Deletion of the C-terminal CBM32 (ΔFHBCBM32 and core variants) completely inactivated the enzyme, whereas the ΔN-CBM variant retained a striking ∼50% of the activity of the wild type. The activity results are in keeping with the mass-spectrometric data, suggesting that the N-terminal deletion did not affect the structural stability of the catalytic domain to the same extent as deletion of the C-terminal CBM32, which probably exposed crucial structural elements of the catalytic domain, making it more susceptible to proteolytic attack. The same pattern was observed when the activity was tested on α-1,2-mannobiose (Supplementary Fig. S4). Hence, the deletions impacted the overall catalytic efficiency rather than local structural elements that could influence the hydrolysis of more complex polysaccharides such as yeast α-mannan. Furthermore, the residual activity (25°C, two days) of all the variants did not change (Supplementary Fig. S6). The inactive variant did not demonstrate any activity on yeast α-mannan, but residual activity was found on α-1,2-mannobiose at high enzyme concentration (1 µ*M*). This is in agreement with the results for alanine and glutamine variants of the general acid Glu533 in *Bt*3990 (equivalent to Glu944 in *Nn*GH92), which substantially impacted the catalytic efficiency against α-1,2-mannobiose but did not completely inactivate the enzyme (Zhu *et al.*, 2010 ▸). Moreover, the core variant did not demonstrate any activity on yeast α-mannan (Fig. 6 ▸ *a*) and α-1,2-mannobiose (Supplementary Fig. S4). This is interesting when compared with *Bt*3990, which consists only of the catalytic core without any accessory domains and exhibits high activity on both yeast α-mannan and α-1,2-mannobiose (Zhu *et al.*, 2010 ▸).

To investigate a possible α-mannan-binding function for the FHB and CBM32 domains, variants were mixed with insoluble yeast cell-wall extracts from *S. cerevisiae* and the amounts of unbound protein were quantified in the supernatant (see Section 2; Fig. 6 ▸ *b*, Supplementary Table S1). Removal of the FHB reduced the population of bound enzyme by ∼50% in comparison to the wild type. Interestingly, C-terminal deletion of both FHB and CBM32 increased the amount of bound enzyme. Considering the absence of CBM32 and its complete loss of activity on all of the substrates tested, including the yeast cell wall (Fig. 6 ▸ *a*, Supplementary Fig. S7), the ΔFHBCBM32 variant showed unusual behaviour. This may suggest that the C-terminal CBM32 serves a role of maintaining the integrity of the *Nn*GH92 architecture, especially the structural elements involved in its catalytic efficiency. Possibly, such an impaired *Nn*GH92 still preserved sufficient structural stability to perform binding, but this is probably much less specific and not limited to α-mannan. This is also observed for the core variant, where a small population of the enzyme was bound to the yeast cell-wall extract (Fig. 6 ▸ *b*). Despite conducting the binding studies at 4°C to limit the activity of the wild type and the variants on insoluble yeast cell wall, there is a risk that the binding moieties were partially hydrolysed by the variants which demonstrated activity on the yeast cell wall (wild type and ΔX216; Supplementary Fig. S7). Therefore, inactive and ΔFHBCBM32 variants might have appeared to show better binding (Fig. 6 ▸ *b*, Supplementary Table S1) because the yeast cell-wall motifs remained intact due to a lack of activity of these variants on the yeast cell wall (Supplementary Fig. S7). Interestingly, similar relative activity of *Nn*GH92 variants was detected towards α-1,2-mannobiose (Supplementary Fig. S4) and yeast α-mannan (Supplementary Fig. S6). ΔFHBCBM32 did not exhibit any activity towards either α-1,2-mannobiose or α-mannan, and hence better binding to the yeast cell wall could be explained by the maintenance of the intact yeast cell-wall motif.

The affinity of the variants for α-mannan was also qualitatively evaluated using native affinity gel electrophoresis (Cockburn *et al.*, 2016 ▸; Supplementary Fig. S8). The electrophoretic mobility was reduced for all of the variants by the presence of soluble α-mannan in the gel matrix. Compared with a control, the movement of the variants was retarded due to binding to the polysaccharide. Due to the large molecular weight of *Nn*GH92 (Table 2 ▸) and the presence of multiple bands in the SDS–PAGE analysis (Supplementary Fig. S1), the influence of the investigated domains was not obvious. However, the ΔFHBCBM32 variant with deletion of the C-terminal CBM32 seemed to regain affinity since the migration of the bands corresponding to this variant in the presence of α-mannan was reduced more than that of the bands corresponding to the ΔFHB variant. This suggests a stronger affinity for ΔFHBCBM32, which is consistent with the binding-affinity results demonstrated on the yeast cell wall (Fig. 6 ▸ *b*).

## 4. Discussion

The study presented here describes structural and biochemical investigation of the *Nn*GH92 α-1,2-mannosidase wild type and domain-deletion variants (Table 2 ▸). The catalytic domains of all of the GH92 α-1,2-mannosidases in the current CAZy database (Lombard *et al.*, 2014 ▸) and wild-type *Nn*GH92 have the same characteristic fold, with the active site having binding residues that are highly conserved in the −1 subsite and more divergent in the +1 subsite, which has previously been proposed to determine the α-mannosidase specificity (Zhu *et al.*, 2010 ▸; Thompson *et al.*, 2018 ▸). The presence of the binding triad Trp477, Glu997 and His996 in the active site of *Nn*GH92 [Trp88, Glu585 and His584 in *Bt*3990 (Zhu *et al.*, 2010 ▸) and Trp70, Glu541 and His540 in *Sp*GH92 (Robb *et al.*, 2017 ▸)], interacting with the mannose residues of the leaving group (+1 subsite) and the biochemical characterization on α-1,2-mannobiose (Kołaczkowski *et al.*, 2022 ▸; Supplementary Figs. S3 and S4) and fungal O-glycans containing mannose residues linked through α-1,2-glycosidic bonds (Kołaczkowski *et al.*, 2022 ▸) confirmed the classification of *Nn*GH92 as a α-1,2-mannosidase. An attempt to solve the crystal structure of the inactive variant (E944Q) to trap α-mannobiose ligands at the active site failed because only poor-quality crystals that were not suitable for diffraction data collection could be grown.

The structure of full-length *Nn*GH92 was solved. The presence of a CBM32 was previously established in the GH92 α-1,2-mannosidases from *Microbacterium* sp. M-90 (aman2; Maruyama *et al.*, 1994 ▸) and *Cellulosimicrobium cellulans* (*Cc*GH92; Tiels *et al.*, 2012 ▸), but none of the studies investigated the structural or biochemical influence of these CBM32s on the enzymes. The absence of CBMs in the GH92 α-mannosidases from *B. thetaiotaomicron* suggests that the presence of a CBM may be driven by the natural habitat of the host organism.

The glycan specificity of CBMs is often guided by the specificity of the catalytic domain to which the CBM is appended (Boraston *et al.*, 2004 ▸). This has been demonstrated for CBM32s connected to the catalytic domains of various GH families (Rao *et al.*, 2006 ▸; Mizutani *et al.*, 2012 ▸; Ficko-Blean *et al.*, 2012 ▸; Newstead *et al.*, 2005 ▸), which are sometimes found in multiple copies within the same enzyme architecture (Abbott *et al.*, 2008 ▸). CBM32 has not been documented to modulate the activity of GH92 α-mannosidases against α-mannans. However, it has been found to increase the activity of the GH5 β-mannanase from *Clostridium thermocellum* against insoluble β-mannans (Mizutani *et al.*, 2012 ▸). Biochemical studies of the *Nn*GH92 variants did not provide an obvious answer to how the appended domains modulate binding to α-manno­oligosaccharides. Since the CBM32 domains were appended to the catalytic domain, the differences in the binding affinity could be hindered by the binding properties of the catalytic domain, especially since CBM32s exhibit low binding affinity (in the millimolar and low micromolar range; Mizutani *et al.*, 2012 ▸; Ficko-Blean & Boraston, 2009 ▸). However, the presence of ManI in the binding site of the N-terminal CBM32, indicated by the overlay with the CBM32 from *Mv*GH33 (Gaskell *et al.*, 1995 ▸), provides the first structural suggestion of CBM32 binding to mannopyranoside rings. This ligand binding by the CBM32 from *Nn*GH92 might not be its only major function. The impaired structural integrity of the catalytic core upon removal of the CBM32s strongly suggests a role of this CBM in protecting structural elements that might be easily accessible for protease attack or simply not fully functional without the appended domains.

This study provides a biochemical and structural investigation of the multi-domain α-1,2-mannosidase *Nn*GH92 that targets yeast α-mannan and fungal protein mannose-rich glycans. Structural comparison to *Bt*3990 confirmed the important amino acids that are involved in the interaction with ManI. A second ManI was bound to the N-terminal CBM32 which allowed the identification of its binding site. This appeared to provide strong evidence for the ability of *Nn*GH92 CBM32 to bind to α-mannooligosaccharides; however, it was not possible to demonstrate this in binding studies. A better binding profile might be obtained by studying these CBM32s expressed separately from the catalytic domain. Understanding the role of the noncatalytic domains is important for the future design of more stable and active bacterial GH92 α-1,2-mannosidases. In particular, *Nn*GH92 can be optimized for efficient enzymatic N- and O-deglycosylation of fungal glycoproteins (Kołaczkowski *et al.*, 2022 ▸).

## 5. Data availability

The data for the GH92 α-1,2-mannosidase from *N. novalis* (*Nn*GH92) have been deposited in the European Nucleotide Archive (ENA) at EMBL–EBI under accession No. LR963497.1 (GenBank sequence ID). The structure and coordinates files for *Nn*GH92 in complex with mannoimidazole have been deposited in the Protein Data Bank under accession code 7nsn. All other data are included in the main article and supporting information.

## 6. Related literature

The following references are cited in the supporting information for this article: Edgar (2004 ▸) and Robert & Gouet (2014 ▸). 

## Supplementary Material

PDB reference: α-1,2-mannosidase from *Neobacillus novalis*, complex with mannoimidazole, 7nsn

Supplementary Figures and Table. DOI: 10.1107/S2059798323001663/jc5054sup1.pdf

## Figures and Tables

**Table 1:** Data-collection and refinement statistics for the *Nn*GH92–ManI complex Values in parentheses are for the outer shell.

Beamline	I04-1, Diamond Light Source
Wavelength (Å)	0.92
Temperature (K)	100
Space group	*P*2_1_
*a*, *b*, *c* (Å)	94.61, 151.94, 114.01
α, β, γ (°)	90, 94.63, 90
Total reflections	410285 (21040)
Unique reflections	141182 (7109)
Completeness (%)	98.1 (99.5)
Multiplicity	2.9 (3.0)
*R*_merge_ (%)	13.2 (77.7)
*R*_meas_ (%)	18.6 (109.0)
*R*_p.i.m._ (%)†	13.1 (76.3)
〈*I*〉/〈σ(*I*)〉	5.1 (1.1)
Resolution range (Å)	47.92–2.29 (2.33–2.29)
CC_1/2_ ‡	0.981 (0.505)
Wilson *B* factor (Å^2^)	24.4
No. of reflections, working set	141151
No. of reflections, test set	6948
Final *R*_cryst_	0.21
Final *R*_free_	0.25
Cruickshank DPI	0.31
No. of non-H atoms	20260
R.m.s. deviations	
Bond lengths (Å)	0.007
Angles (°)	1.484
Average *B* factors (Å^2^)	
Chain *A*, protein	31
MVL-1	27
MVL-2	36
Chain *B*, protein	36
MVL-1	23
*MolProbity* score	2.09
Ramachandran plot	
Most favoured (%)	96.25
Outliers (%)	0.15
PDB code	7nsn

† *R*_p.i.m._ = 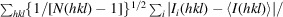 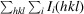 .

‡ CC_1/2_ is defined in Karplus & Diederichs (2012 ▸).

**Table 2:** Wild-type *Nn*GH92 and the truncated variants investigated in this study

*Nn*GH92 variant name	CBM-like domain†	N-terminal CBM32†	C-terminal CBM32†	FHB†	MW‡ (kDa)	*T*_m_ § (°C)	Relative activity¶ (%)
Wild type (LR963497.1††)	+	+	+	+	151.7	60	100
ΔFHB	+	+	+	—	141.9	53	81 ± 0.7
ΔFHBCBM32	+	+	—	—	125.2	48	11 ± 2.0
ΔN-CBM	—	—	+	+	112.8	50	55 ± 2.0
Core	—	—	—	—	85.5	49	4 ± 0.4
Inactive (E944Q)	+	+	+	+	151.7	60	4 ± 0.5

† +, present; −, truncated.

‡ Theoretical molecular weight.

§ Melting temperature at pH 6.

¶ Activity measured on yeast α-mannan and calculated using the wild type as the reference (Fig. 6 ▸); the error corresponds to the standard deviation of triplicate sample reactions.

†† European Nucleotide Archive accession number.
