# Supplementary material for: Structural and functional characterization of a multi-domain GH92 α-1,2-mannosidase from Neobacillus novalis
Source: Acta Crystallogr D Struct Biol. 2023 Apr 18;79(Pt 5):387–400. doi: 10.1107/S2059798323001663 (PMC10167667; doi:10.1107/S2059798323001663)
Supplement: Supplementary file 1 [file d-79-00387-sup1.pdf]

Acta Cryst D STRUCTURAL BIOLOGY

Volume 79 (2023)

Supporting information for article:

Structural and functional characterization of a multi-domain GH92 α-1,2-mannosidase from *Neobacillus novalis*

Bartlomiej M. Kolaczkowski, Olga V. Moroz, Elena Blagova, Gideon J. Davies, Marie Sofie Møller, Anne S. Meyer, Peter Westh, Kenneth Jensen, Keith S. Wilson and Kristian B. R. M. Krogh

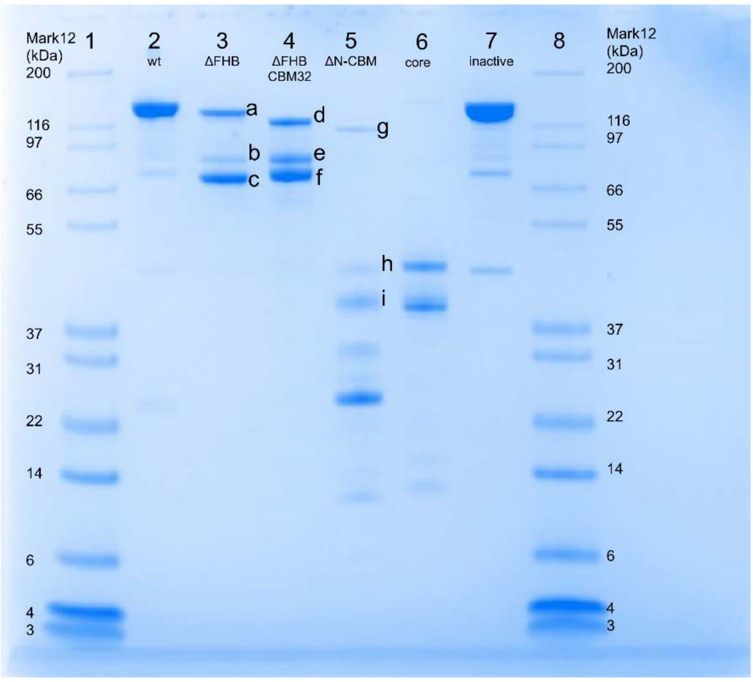

Figure S1 SDS-PAGE gel of the purified *Nn*GH92 wild-type and variants used in this study. Lane 1, 8: Molecular weight standard (Mark12, Invitrogen); lane 2: *Nn*GH92 wild-type; lane 3: ΔFHB; lane 4: ΔFHBCBM32; lane 5: ΔN-CBM32, lane 6: core; lane 7: inactive (E944Q) The gel was stained with Coomassie Blue and 1 µg of each enzyme was loaded on the gel. The bands indicated by the letters (a-i) were excised and digested with trypsin. The isolated peptides were analyzed with mass spectrometry and matched with the protein sequence of the corresponding *Nn*GH92 variants. The bottom bands (b, c, e, f) corresponding to the C-terminal deletion variants (ΔFHB and ΔFHBCBM32), were randomly truncated from the protein C-terminus with poor coverage in the catalytic domain region. The bottom bands (h, i) of the N-terminal deletion variant (ΔCBM) maintained good protein coverage within the catalytic domain. No common cleavage pattern was identified.

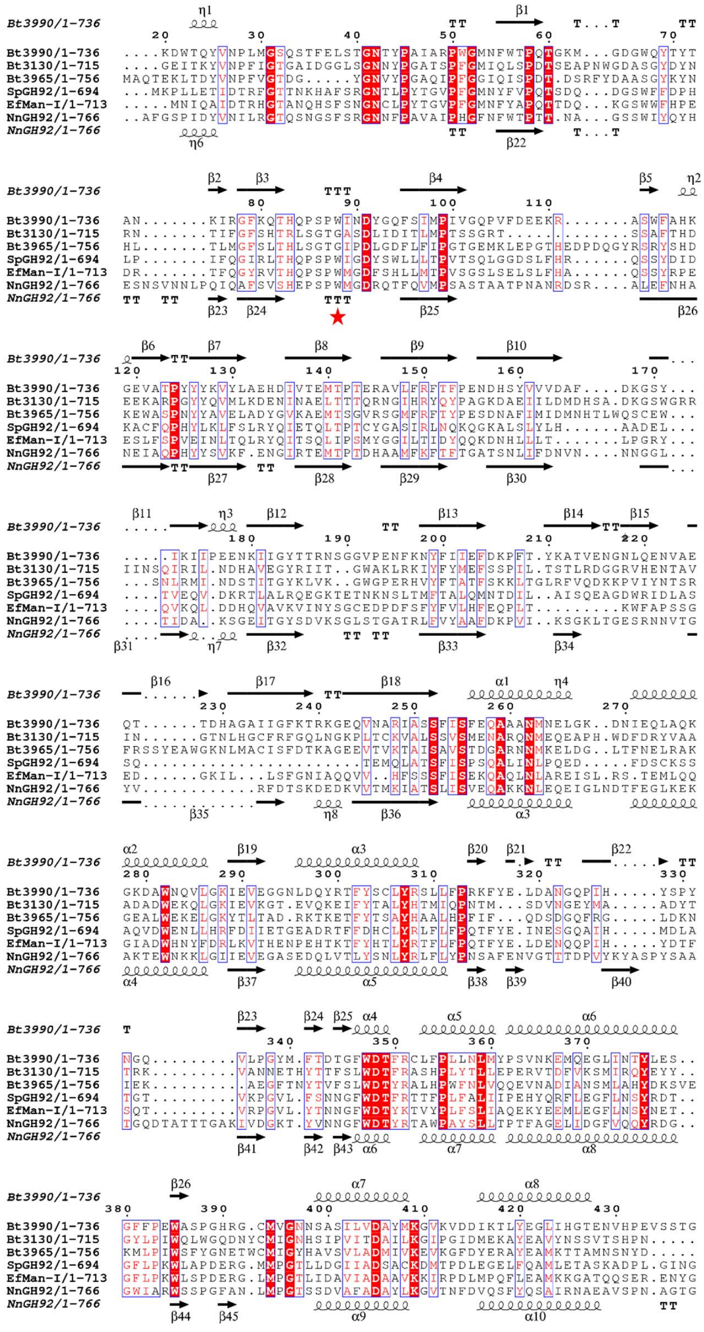
Bt3990/1-736
Bt3130/1-715
Bt3965/1-756
SpGH92/1-694
EfMan-I/1-713
NnGH92/1-766

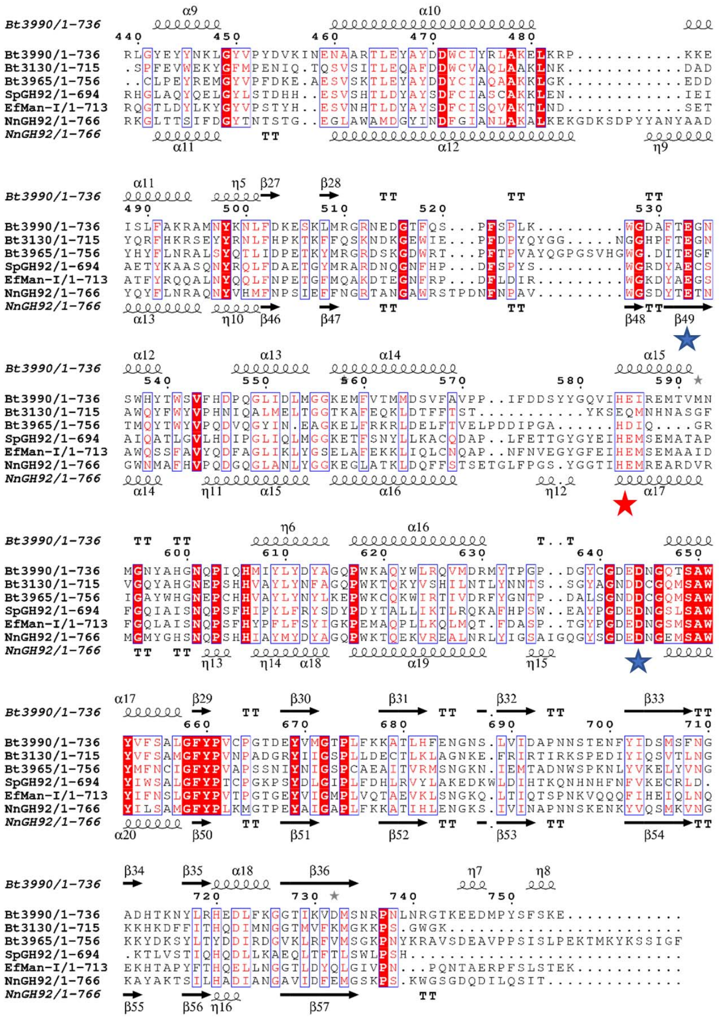

Figure S2 Multiple sequence alignment of catalytic domains from 6 GH92 α-mannosidases with experimentally determined structures. Secondary structure elements for *Bt*3990 (PDB 2WZS) and *Nn*GH92 (PDB: 7NSN) are shown on top and bottom of the alignment, respectively. The numbering is based on the sequence of *Bt*3990. The general acid (E533) and Brønstead base (D644) are marked with a blue star whereas the residues driving specificity of α-1,2-mannosidase are marked with a red star (W88, H584-E585). Identical residues are marked in white characters on a red background. Highly similar residues are framed in a blue box. The alignment was created with MUSCLE algorithm (Edgar, 2004) and the figure prepared using ESPript 3.0 web server with default parameters (Robert & Gouet, 2014).

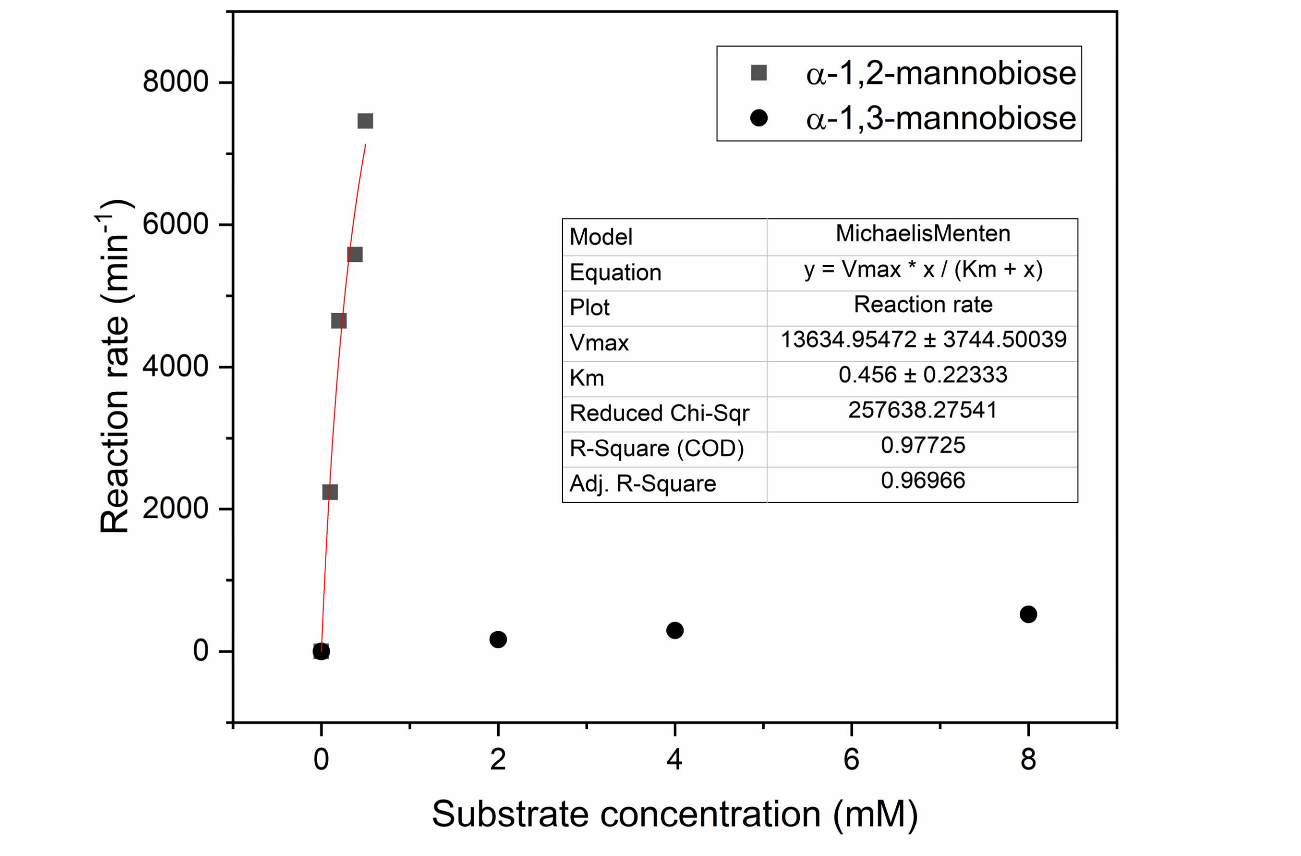

Figure S3 *N*nGH92 Michaelis-Menten kinetics on α-1,2-mannobiose and α-1,3-mannobiose. Each dot represents initial hydrolysis rate measured at different concentrations of substrates. Mannose release was quantified by Megazyme International kit for D-mannose assay kit using mannose standard curve. Solid lines for α-1,2-mannobiose represent a non-linear fit of Michaelis-Menten equation.

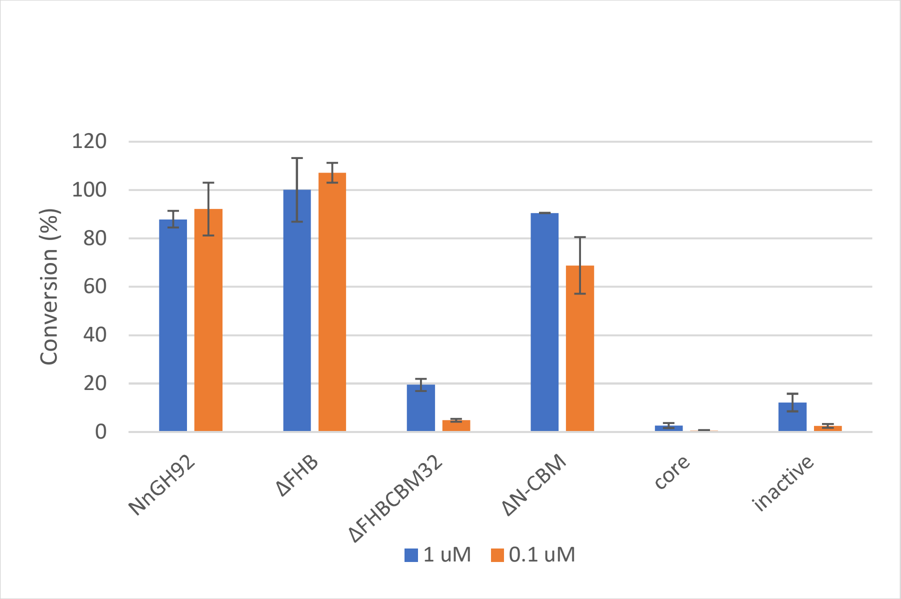

Figure S4 Activity profile of the *Nn*GH92 variants on 1.46 mM α-1,2-mannobiose dissolved in the assay buffer.

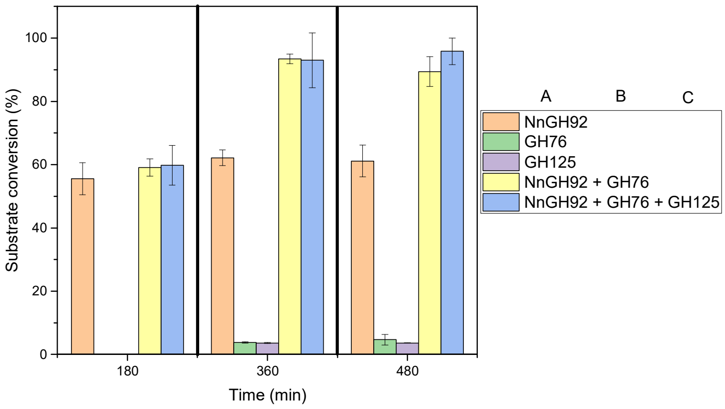

Figure S5 Yeast α-mannan hydrolysis by *Nn*GH92 (E_0 = 0.13 μM), the GH76 endo-α-1,6-mannanase (E_0 = 0.03 μM) and the GH125 exo-α-1,6-mannosidase (E_0 = 0.34 μM). The yeast α-mannan (S_0 = 2.5 mg/mL) was dissolved in the assay. The reaction was initiated by adding the enzymes in the order of t1=0 min (A), t2=180 min (B), t3=360 min (C), as shown in the figure legend. The blank spaces under the corresponding letter indicate the addition of an equivalent volume of the assay buffer. The extent of hydrolysis was followed by reducing sugar assay (PAHBAH).

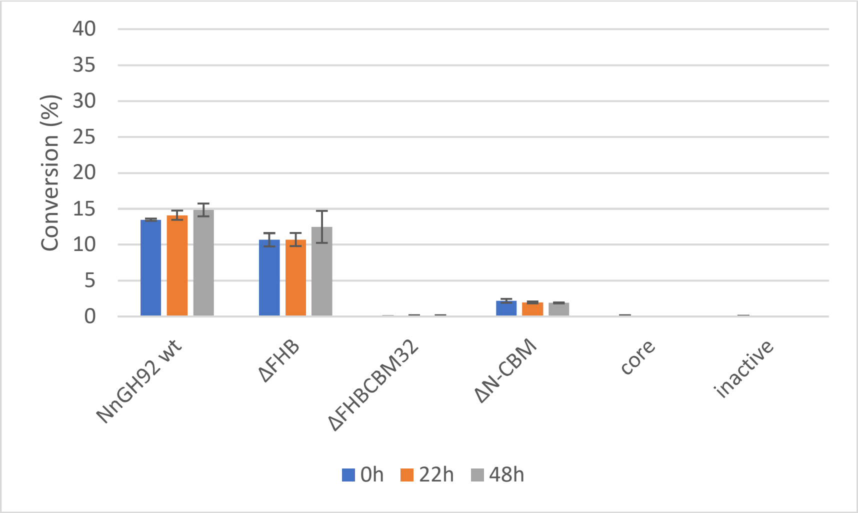

Figure S6 Residual activity of the *Nn*GH92 variants towards yeast alpha-mannan. The enzymes (0.1 µM) were incubated for 0h, 22h and 48h at 25°C. Then at each time point, enzymes were mixed with 2.5 g/L α-mannan in assay buffer and incubated for 15 min. The enzyme activity was verified using reducing sugar assay (PAHBAH). No change in the residual activity for each tested enzyme was observed.

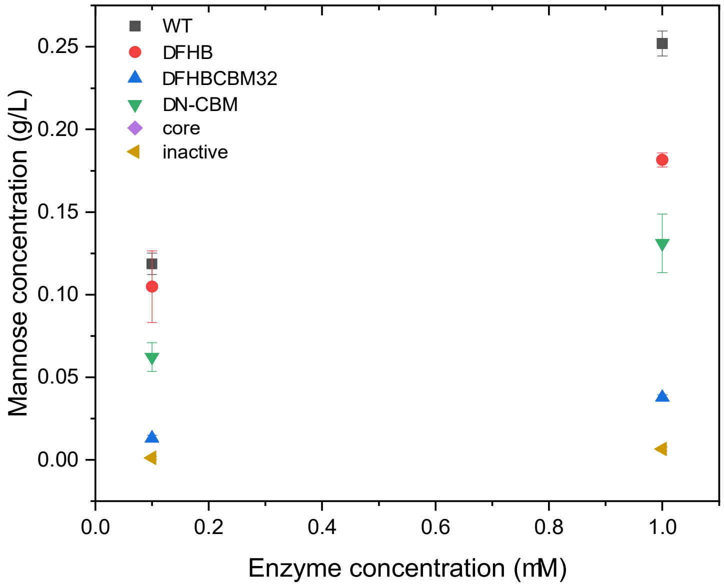

Figure S7 Activity profile of the *Nn*GH92 variants (1 h, 37°C) on the yeast cell wall (60 g/L dry weight cell in assay buffer) extracted from *S. cerevisiae*.

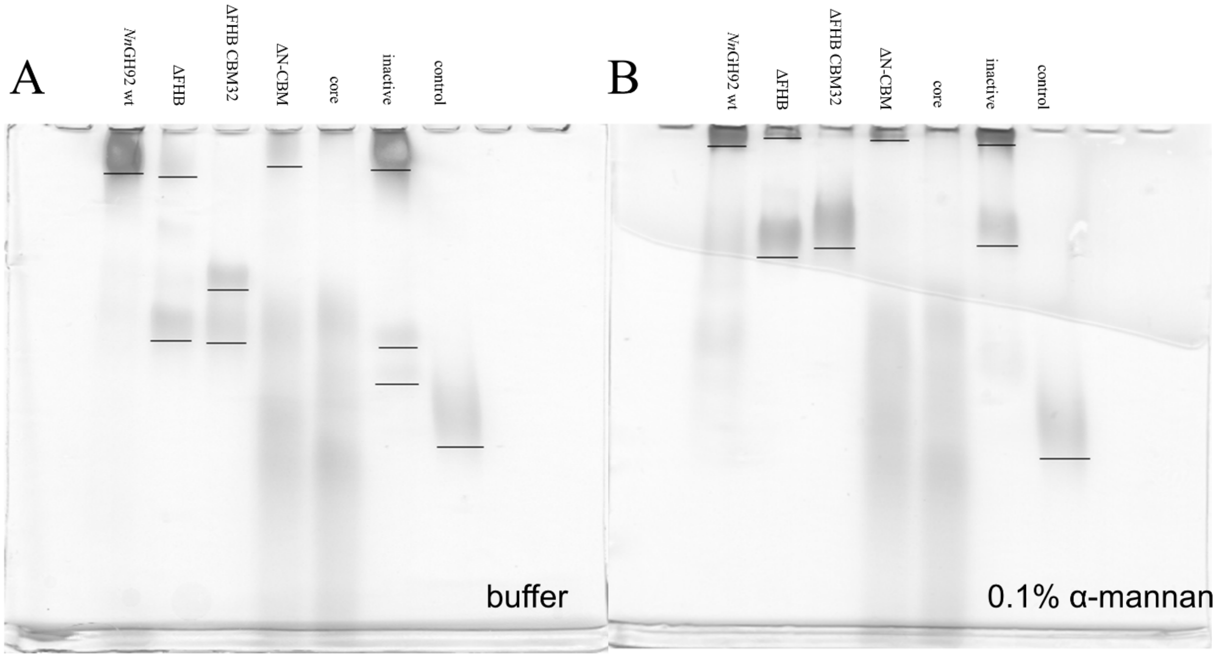

Figure S8 Native affinity gel electrophoresis of the *Nn*GH92 wild-type and variants. The enzymes were run in the control gel (A) and in the gel with yeast α-mannan (B). The migration of the bands corresponding to the enzymes were compared between two gels. The lane 'control' contained GH125 α-1,6-mannosidase used as a control protein due to it is high molecular weight.

Table S1 Derived parameters from the binding isotherm of *Nn*GH92 variants in Figure 6B.

The ± values correspond to the error of non-linear fit of binding isotherm curves.

***Nn*GH92**	Γ_{max} µmol/g	K_d µM	**Statistics** Adj. R-Square
wild-type	0.29 ± 0.02	2.74 ± 0.42	0.99
ΔFHB	0.13 ± 0.01	1.16 ± 0.48	0.91
ΔFHBCBM32	0.24 ± 0.01	0.38 ± 0.10	0.97
inactive	0.28 ± 0.04	0.47 ± 0.25	0.87

Edgar, R. C. (2004). *Nucleic Acids Res.* **32**, 1792–1797.

Robert, X. & Gouet, P. (2014). *Nucleic Acids Res.* **42**, 320–324.
